# Postpartum depression and associated factors among postpartum women in Ethiopia: a systematic review and meta-analysis, 2020

**DOI:** 10.1186/s40985-020-00136-3

**Published:** 2020-09-16

**Authors:** Tadesse Tolossa, Getahun Fetensa, Mekdes Tigistu Yilma, Muktar Abadiga, Bizuneh Wakuma, Merga Besho, Ginenus Fekadu, Werku Etafa

**Affiliations:** 1 Department of Public Health, Institutes of Health Sciences, Wollega University, Nekemte, Ethiopia; 2 Department of Nursing, School of Nursing and Midwifery, Wollega University, Nekemte, Ethiopia; 3 Department of Midwifery, School of Nursing and Midwifery, Wollega University, Nekemte, Ethiopia; 4 Department of Pharmacy, Institutes of Health Sciences, Wollega University, Nekemte, Ethiopia

**Keywords:** Postpartum depression, Systematic review, Ethiopia

## Abstract

**Introduction:**

The postpartum period is recognized as a high-risk period for the development of various mood disorders like postpartum depression. Globally, postnatal depression is a serious public health problem that has a negative impact on the mother’s health and child development, especially in developing countries. In Ethiopia, even though there are different primary studies conducted on postpartum depression, there is no nationally representative evidence. Therefore, the aim of this systematic review and meta-analysis was to estimate the pooled prevalence and associated factors of postpartum depression in Ethiopia.

**Methods:**

Published and unpublished articles from various electronic databases and digital libraries were accessed. This systematic review included studies that were conducted on the magnitude and factors associated with postpartum depression among postnatal women in Ethiopia. A random-effect model was used to estimate the pooled magnitude of postpartum depression with a 95% confidence interval (CI). Inverse variance (I^2^) was used to visualize the presence of heterogeneity, and forest plot was used to estimate the pooled magnitude of postpartum depression. Publication bias was assessed by funnel plots and Egger’s statistical tests. A meta-regression and subgroup analysis were computed to minimize underlying heterogeneity.

**Result:**

Initially, a total of 764 studies were accessed. Twenty-eight full articles were assessed for eligibility criteria, of which twelve studies fulfilled inclusion criteria were included in the final meta-analysis. The overall pooled magnitude of postpartum depression was 22.89% (95% CI 17.75%, 28.03%) with the lowest (12.20%) and highest (33.82%) in the Southern nations region. Unplanned pregnancy, domestic violence, lack of social support, previous history of depression, infant loss, and dissatisfaction in marriage showed a statistically significant association with postpartum depression.

**Conclusions:**

In the current analysis, the prevalence of postpartum depression was high as compared with other developing countries. Routine screening of mothers in the postpartum period and integrating mental health with maternal health care is highly recommended.

## Introduction

The postpartum period is recognized as a high-risk period for the development of various mood disorders which include postpartum blue, major depression, and postpartum psychosis [1]. According to the standard diagnostic and statistical manual of mental disorders (DSM-5), postpartum depression (PPD) is one type of depressive disorder that occurs during pregnancy or within 4 weeks after childbirth [2, 3]. The International Classification of Diseases 10 (ICD-10) also recognizes postpartum depression as syndromes associated with pregnancy or the puerperium that involve significant mental and behavioral features. According to the ICD-10 classification, PPD is characterized by a period of depressed mood lasting at least 2 weeks accompanied by other symptoms such as difficulty concentrating, feelings of guilt, hopelessness, recurrent thoughts of suicide, changes in appetite or sleep, psychomotor agitation, and reduced energy [4].

Literature has shown that the magnitude of PPD varies from country to country and region to region. The prevalence of postpartum depression is lower in women from Europe, Australia, and the USA than women from Asia and South Africa [5]. It is estimated that the prevalence of PPD is about 10% in developed countries [6] and nearly 20% in developing countries [7]. The magnitude of PPD is 6.7% in China [8], 21% in Japan [9], 11 to 16% in India [10], and 39.4% in Bangladesh [11]. In Africa, the prevalence of PPD is 18.7% in Kenya [12], 7% in Ghana [13], and 9.2% in Sudan [14]. In Ethiopia, studies conducted in different parts of the country showed that the magnitude of postpartum depression ranges from 12.2 [15] to 33.82% [16].

Postpartum depression affects the welfare of mothers, partners, family members, and the growth and development of babies [17]. In addition, it affects mother’s ability of parenting their children. Postpartum women shows hostile and disengaged parenting behavior, and decreases mothers’ caregiving ability for infants [18].

Different studies indicate that the development of postpartum depression leads to reduced infant growth and malnutrition due to early weaning and poor infant feeding practices [19, 20]. Furthermore, due to its occurrence at a crucial period for an infant’s physical, psychological, and mental development, it causes children to have cognitive, emotional and behavioral problems [21].

Studies have shown that factors such as low monthly income, age under 24 years, unemployment due to pregnancy, dissatisfaction in marital condition, and intimate partner violence were associated with postpartum depression [11, 12, 14, 16, 22]. Obstetric-related factors such as unplanned pregnancy, history of miscarriage/stillbirth, and being the first-time mother were associated with postpartum depression [11, 22, 23]. Social and behavioral factors such as having a history of substance use, poor social support, history of previous depression, and experiencing the death of infants were also some of the factors associated with postpartum depression [16, 22, 23].

In developing countries including Ethiopia, PPD is often neglected in the health care system, and there is no screening during postnatal follow-up and no referral of PPD cases for appropriate mental health services [24]. Despite the wide-ranging variations in the prevalence and associated factors of postpartum depression, there is no review conducted with respect to estimating postpartum depression in Ethiopia. Thus, reliable estimates of postpartum depression are required for the development of national and international health policies to improve maternal mental health services. Therefore, the aim of this systematic review and meta-analysis was to estimate the pooled prevalence of postpartum depression and its associated factors among postpartum women in Ethiopia.

## Methods

### Search strategy

In this review, two investigators (TT and GF) independently searched six electronic databases such as Medline, Pub Med, Cochrane library, the Web of Science, Google Scholar, and Scopus using the key terms “postpartum depression”, “postpartum stress”, and “postpartum distress” combined with “postpartum women”, and “Ethiopia”. Addis Ababa University Digital Library and African digital library were searched to find unpublished papers [25–28]. Literature was downloaded to Endnote (version X7) to maintain and manage citations and facilitate the review process. The existence of similar systematic reviews and meta-analysis was checked to avoid duplication. The search was conducted from December 1, 2019 to January 1, 2020, and the meta-analysis is reported according to the PRISMA guideline.

The predefined search terms were used to allow us a comprehensive search of important studies included in our review. All fields within records and Medical Subject Headings (MeSH terms) were used to help enlarge the search in advanced PubMed search. The following search strategies were adapted for the various databases using the two important Boolean operators and search engines with initial search terms (“prevalence of postpartum depression” OR “magnitude of postpartum depression” AND “associated factors of postpartum depression” OR “determinants of postpartum depression” AND “pregnant women” AND “Ethiopia”). The reference lists of identified studies were also reviewed to find additional articles.

### Selection and eligibility criteria

#### Inclusion criteria

This systematic review included studies that were conducted on the prevalence and determinants of postpartum depression among postnatal women in Ethiopia.

- Participants: Women in the postpartum period
- Study design: Observational studies
- Language: English language exclusively
- Setting: Studies, which were conducted in Ethiopia, were included in the review.
- Publication: All published and unpublished studies were included.
- Sample size: Any

#### Exclusion criteria

Conference abstracts and non-human studies were excluded. We tried to contact the primary authors of the articles with incomplete information, and we excluded articles that were not accessible after contacting the principal investigator two times via email.

### Outcome measurement

This study has two main outcomes. The first outcome was to estimate the prevalence of postpartum depression among postpartum women. It was calculated as the number of women with postpartum depression divided by the total number of women in the postpartum period multiplied by 100. Postpartum depression was assessed by using different measurement scales such as an Edinburgh Postnatal Depression Scale (EPDS), Patient Health Questionnaire scale, and Kessler 10 scale (K10). EPDS has 10 items, and each item has 4 Likert; it has a maximum score of 30 and minimum score of zero. The outcome variable was categorized into EPDS ≥ 10 which indicates clinically significant depressive symptoms and EPDS < 10 which does not indicate clinically significant depressive symptoms [29]. The Patient Health Questionnaire scale has 9 items and a total sum score of 27. The score ≥ 10 indicates clinically significant depressive symptoms and a total sum score < 10 indicates the absence of clinically significant depressive symptoms [30]. The K10 scale involves 10 questions about emotional states each with a five-level response scale. Each item is scored from one “none of the time” to five “all of the time”. Scores of the 10 items are then summed, yielding a minimum score of 10 and a maximum score of 50. Low scores indicate low levels of psychological distress, and high scores indicate high levels of psychological distress [31].

The second outcome of this study was the determinants of postpartum depression among postnatal women. Data for this outcome were extracted in a format of two by two tables on the Microsoft Excel spreadsheet, and then the log odds ratio for each factor was calculated based on the findings of the original studies. Determinants included in this study were pregnancy intention (planned versus unplanned), type of delivery (normal vaginal delivery versus instrumental or caesarean delivery), social support (having social support versus lack of social support), previous history of the death of infant (absence versus presence), domestic violence (absence versus presence), previous history of depression (absence versus presence), and marital satisfaction (satisfied versus not satisfied).

### Quality assessment and data extraction

The Joanna Briggs Institute Meta-Analysis of Statistics Assessment and Review Instrument (JBI-MAStARI) was used for critical appraisal. Initially, the reference management software (Endnote version X7) was used to combine database search results and to remove duplicate articles manually. Then, the titles and abstracts of the studies were exhaustively assessed based on the relevance of the outcome. Full text of the remaining articles were evaluated for eligibility based on prearranged inclusion and exclusion criteria. Finally, studies that had scored 7 and above on the JBI quality appraisal checklist were included in the systematic review and meta-analysis. The checklist for data extraction comprises the name of authors, publication year, region (the area where the study was conducted), study design, the setting of the study (community-based or institution-based), sample size, response rate, and participants with the outcome (Table 1). Two reviewers (TT and GF) extracted data using a standardized data extraction checklist on the Microsoft excel spreadsheet. Incongruities between two independent reviewers were compromised by involving a third reviewer (MA).

**Table 1:** Summary of included studies regarding the postpartum depression and its determinants among postnatal women in Ethiopia, 2020

S.N	Primary author	Year	Study period	Region	Outcome measurement	Study design	Study setting	Sample size	Response rate	Number of PPD cases	Magnitude of PPD (95% CI)
1	Abayneh S et al. [32]	2016	February–March 2016	Harar	EPDS	Cross-sectional	Institution based	122	100%	16	13.11 (7.12, 19.1)
2	Addishiwet F et al. [33]	2018	January–Feb 2017	AA	EPDS	Cross-sectional	Institution based	618	97.60%	144	23.30 (19.90, 26.63)
3	Habrasa T [25]	2016	April 15–April 30, 2015	AA	Kessler-10 scale	Cross-sectional	Institution based	295	100%	82	27.80 (22.68, 32.91)
4	Addishiwet F et al. [34]	2018	March–April 2016	AA	EPDS	Cross-sectional	Institution based	618	98.20%	144	23.30 (19.90, 26.63)
5	Muktar A [22]	2019	May 15–June 5, 2019	Oromia	EPDS	Cross-sectional	Community base	287	97%	60	20.91 (16.20, 25.61)
6	Tigistu T et al. [23]	2018	March 15–April 15, 2017	SNNP	PHQ-9	Cross-sectional	Community base	456	99.00%	102	22.37 (18.54, 26.19)
7	Sitotaw K et al. [16]	2017	December 1–February 1, 2017	SNNP	EPDS	Cross-sectional	Institution based	408	97%	138	33.82 (29.23, 38.41)
8	Amsale A et al. [35]	2019	May to June 2018	Amhara	EPDS	Cross-sectional	Institution based	511	97%	113	22.11 (18.52, 25.71)
9	Ashish K et al. [36]	2019	Not stated	National level	Self-reported questionnaire	Cross-sectional	Community base	2000	100%	660	33.00 (30.93, 35.06)
10	Solomon S et al. [37]	2019	February 1–March 2, 2018	Amhara	EPDS	Cross-sectional	Community base	596	97.4%	141	23.65(20.24, 27.07)
11	Telake Azale et al. [15]	2018	March and June 2014	SNNP	PHQ-9	Cross-sectional	Community base	3147	100%	385	12.23 (11.08, 13.37)
12	Deribachew H et al. [38]	2016	Not stated	Tigrai	EPDS	Cross-sectional	Community base	616	97.3%	117	18.99 (15.89, 22.09)

*AA* Addis Ababa, *CI* confidence interval, *EPDS* Edinburgh Postnatal Depression Scale, *PPD* postpartum depression, *SNNP* Southern Nation Nationalities and People, *PHQ-9* Patient Health Questionnaire

### Statistical analysis and data synthesis

Data were retrieved on a Microsoft Excel spreadsheet format and imported to STATA version 14 statistical software for analysis. The logarithm and standard error of the odds ratio (OR) for each included study were generated using the “generate” command on STATA. The pooled magnitude of postpartum depression and its determinants was presented in the form of a forest plot. The presence of heterogeneity among the included studies was checked by Cochran’s Q test (reported as the *P* value) and inverse variance index (I^2^). A random effects model was computed to estimate the pooled prevalence of postpartum depression as a high degree of heterogeneity was observed. Meta-regression was conducted to identify the source of heterogeneity by using sample size and year of publication, but none of the variables showed significant presence of heterogeneity. Subgroup analysis was conducted using region and setting of the study to minimize potential variability between included studies. A funnel plot was used to check the presence of publication bias. Furthermore, Egger and Begg’s statistical test was used to check the statistical significance of publication bias. A random effects model was used for the second outcome as a moderate-to-high degree of heterogeneity was observed among five variables (pregnancy intention, social support, previous history of the death of an infant, previous history of depression, and marital satisfaction). In two variables (a type of delivery, domestic violence), a fixed effects model was used as there was no heterogeneity among included studies.

## Result

### Search result

**Fig. 1:**
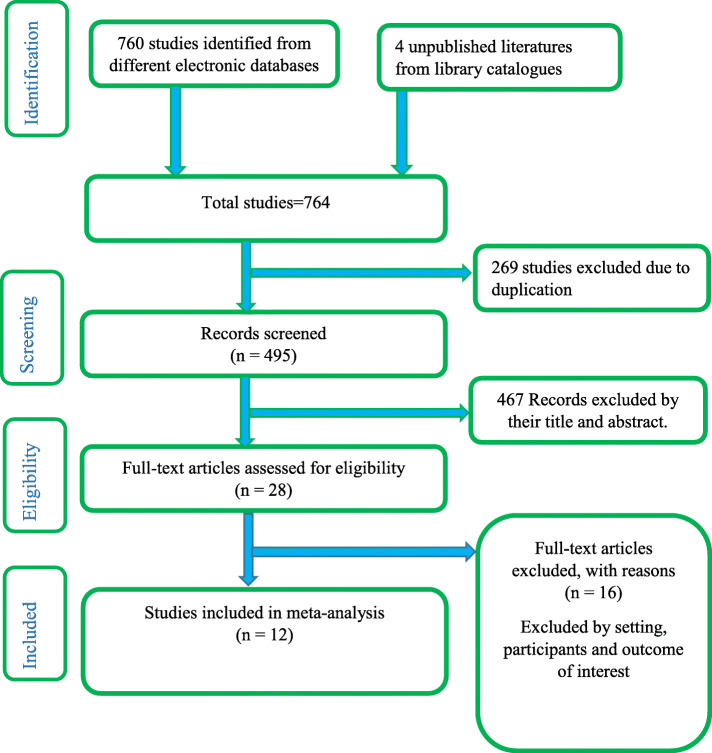
Flow diagram of the studies included in the meta-analysis

Initially, 764 studies were accessed from different electronic database searches and digital library catalogs, of which 269 studies were removed due to duplicates. Then, the titles and abstracts of remaining studies were assessed, and 467 studies were screened and excluded due to non-relevance to the study. The remaining 28 full-text articles were evaluated for eligibility based on the predetermined inclusion and exclusion criteria. Finally, twelve articles that fulfilled the eligibility criteria were included in the final analysis (Fig. 1).

### Overview of included studies

Of the twelve included studies, three were conducted in Addis Ababa [25, 33, 34], three in Southern nation, nationalities and peoples (SNNP) region [15, 16, 23], one in Oromia [22], two in Amhara [35, 37], one in Harari regional state [32], one in Tigrai region [38], and one study at national level [36]. No studies were reported from Benishangul Gumuz, Afar, Somali, and Gambela region. The sample size ranges from a minimum of 122 from the Harari region [32] to a maximum of 3147 from SNNP [15]. In a current meta-analysis, 9674 women in the postpartum period were included to estimate the pooled prevalence of postpartum depression in Ethiopia. All of the articles included in this study were published between 2016 [25] and 2019 [22, 35–37]. Eleven of the included studies were published in peer-reviewed journals, while one was an unpublished master thesis at Addis Ababa University [25]. All the studies selected in this review were cross-sectional in design. Six studies were conducted at the institution level [16, 25, 32–35], and six were community-based studies [15, 22, 23, 36–38]. Regarding the response rate, almost all studies had a good response rate (> 85%) (Table 1). Determinants included in this study were pregnancy intention, type of delivery, social support, previous history of the death of an infant, domestic violence, previous history of depression, and marital satisfaction.

### The prevalence of postpartum depression

**Fig. 2:**
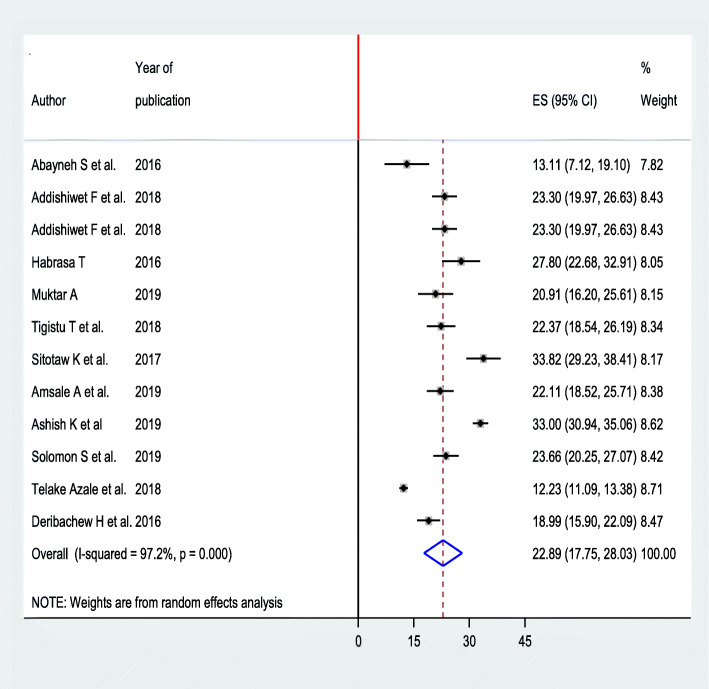
Forest plot for pooled prevalence of postpartum depression among postnatal women in Ethiopia, 2020

The highest prevalence of postpartum depression observed among postnatal women of Mizan Tepi hospital Southwest Ethiopia was 33.82% (95% CI 29.23, 38.41) [16], while the lowest was 12.23% from the same region [15]. The I^2^ test result indicates high heterogeneity (*I*^2^ = 97.2%), which is suggestive of using random effects model. The overall pooled magnitude of postpartum depression in Ethiopia was 22.89% (95% CI 17.75%, 28.03%) (Fig. 2).

Meta-regression was employed to identify the potential sources of heterogeneity by using sample size and year of publication, but none of these variables were found to be statistically significant.

**Fig. 3:**
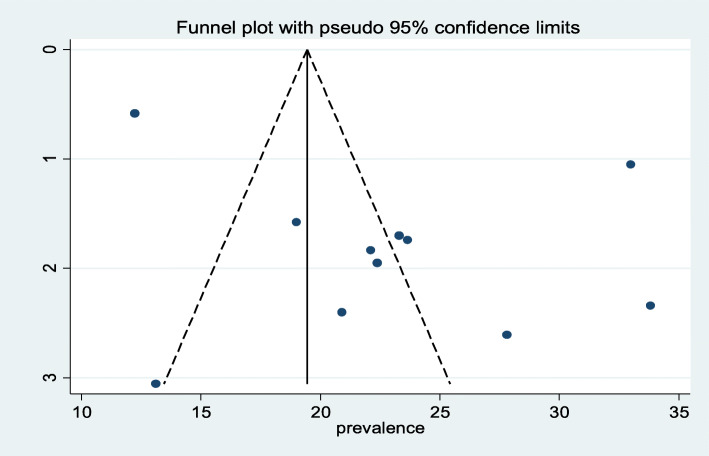
Funnel plot with 95% confidence limits of the pooled prevalence of postpartum depression among postnatal women in Ethiopia, 2020

The traditional graphical funnel plot was asymmetric showing the presence of publication bias (Fig. 3). In addition, Egger’s weighted and Begg’s tests were computed to see the significant presence of publication bias. Both tests showed no statistically significant presence of publication bias (*P* = 0.07, *P* = 0.945), respectively.

### Subgroup analysis

**Fig. 4:**
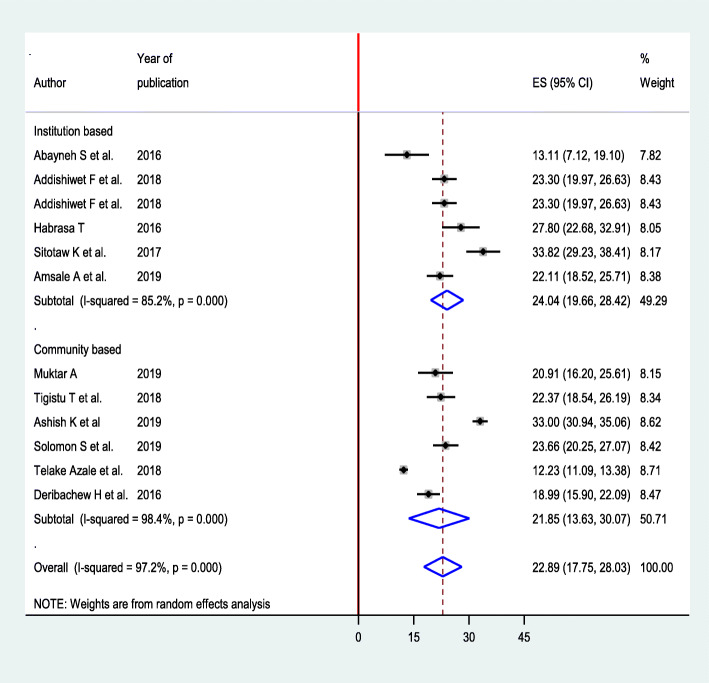
Subgroup analysis based on the setting for prevalence of postpartum depression among postnatal women in Ethiopia, 2020

Subgroup analysis was performed by setting to minimize potential variability between studies. Accordingly, the prevalence of postpartum depression was high at institution level 24.04 (95% CI 19.66, 28.42) and low in community-based studies 21.85 (95% CI 13.63, 30.07) (Fig. 4).

### Sensitivity analysis

**Fig. 5:**
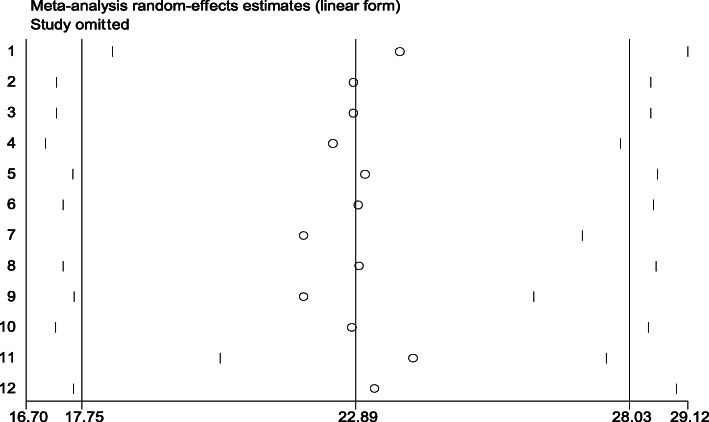
Sensitivity analysis for the prevalence of postpartum depression among postnatal women in Ethiopia, 2020

To identify outliers of a single study influence on the overall meta-analysis, a sensitivity analysis was performed using a random effects model, and the result showed that there was no strong evidence for the effect of single study on the overall meta-analysis result (Fig. 5).

### Factors associated with postpartum depression

#### Association between pregnancy intention and postpartum depression

**Fig. 6:**
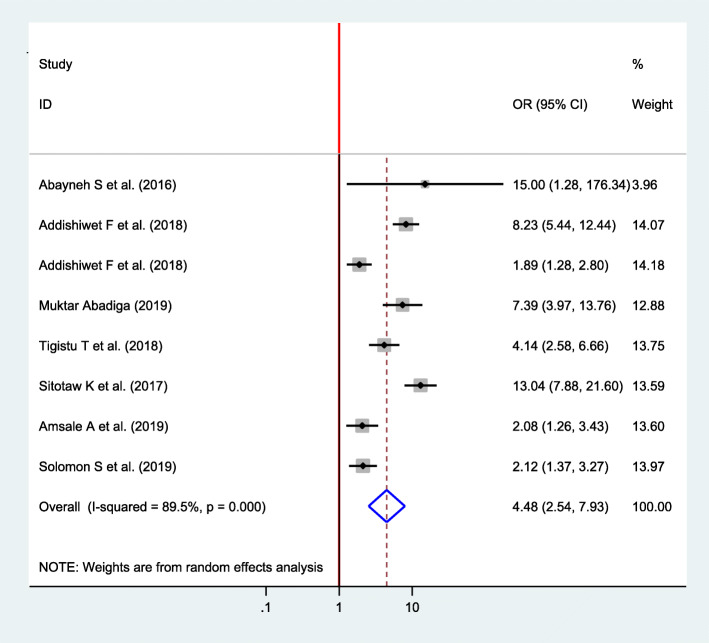
Forest plot for pooled association between pregnancy intention and postpartum depression in Ethiopia, 2020

From a total of twelve included studies, eight studies were selected to identify the association between pregnancy intention and postpartum depression [16, 22, 23, 32–35, 37]. There was a significant association between pregnancy intention and postpartum depression among all included studies. The pooled finding showed that the odds of postpartum depression were 4.48 times higher among women who had unplanned pregnancy as compared with women with planned pregnancy (OR = 4.48, 95% CI 2.54, 7.93) (Fig. 6).

#### Association between type of delivery and postpartum depression

**Fig. 7:**
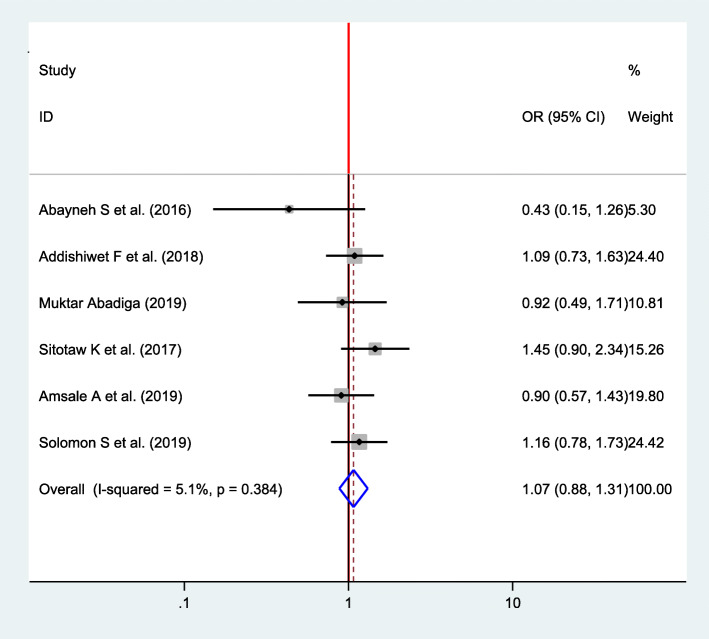
Forest plot for pooled association between type of delivery and postpartum depression in Ethiopia, 2020

Six studies were included to compute the association between a type of delivery and postpartum depression for final meta-analysis [16, 22, 32, 33, 35, 37]. A fixed effects model was used to estimate the pooled association between type of delivery and postpartum depression (*I*^2^ = 5.1, *P* value = 0.384). The pooled result of the analysis indicates that there was no statistically significant association (OR = 1.07, CI 0.88, 1.31) (Fig. 7).

#### Association between domestic violence and postpartum depression

**Fig. 8:**
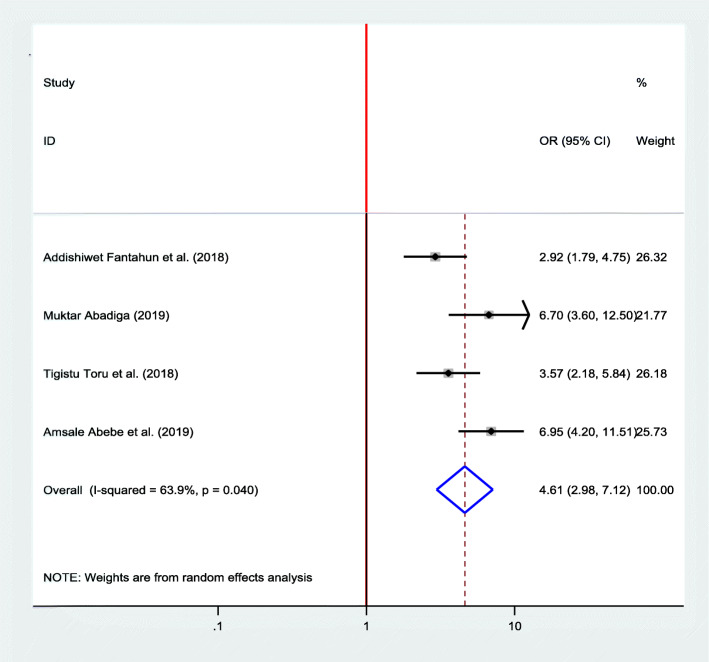
Forest plot for pooled association between domestic violence and postpartum depression in Ethiopia, 2020

Four studies [22, 23, 33, 35] were included in the meta-analysis to see the association between domestic violence and postpartum depression, and all of them showed a significant association. A random effects model was used as high heterogeneity was observed (*I*^2^ = 63.9, *P* value = 0.04). The pooled finding indicated that women who experienced domestic violence were 4.61 more likely to develop postpartum depression as compared with their counterparts (OR = 4.61, 95% CI 2.98, 7.12) (Fig. 8).

#### Association between the previous history of depression and postpartum depression

**Fig. 9:**
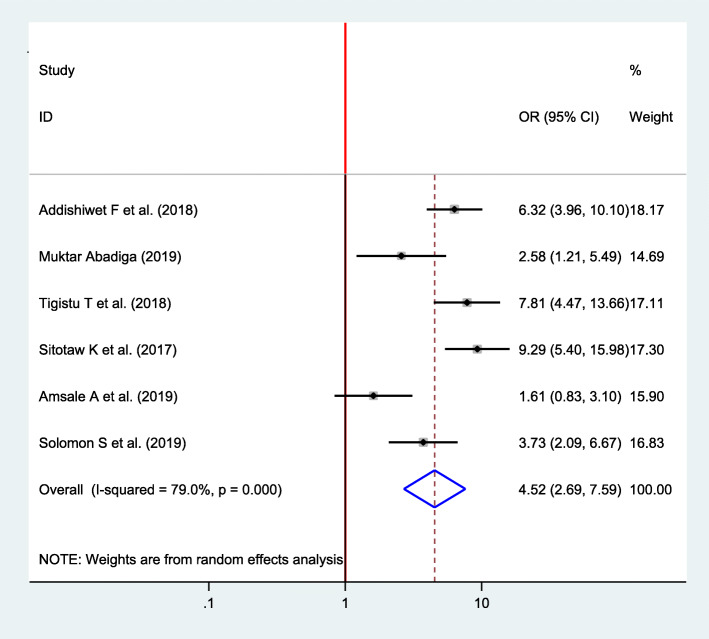
Forest plot for pooled association between previous history of depression and postpartum depression in Ethiopia, 2020

Six studies were included to assess the association between the previous history of depression and postpartum depression [16, 22, 23, 33, 35, 37]. Except for one study, all of them have showed a significant association between the previous history of depression and postpartum depression [35]. The pooled analysis revealed that the odds of developing postpartum depression were 4.52 times higher among women who had the previous history of depression than their counterparts (OR = 4.52, 95% CI 2.69, 7.59) (Fig. 9).

#### Association between social support and postpartum depression

**Fig. 10:**
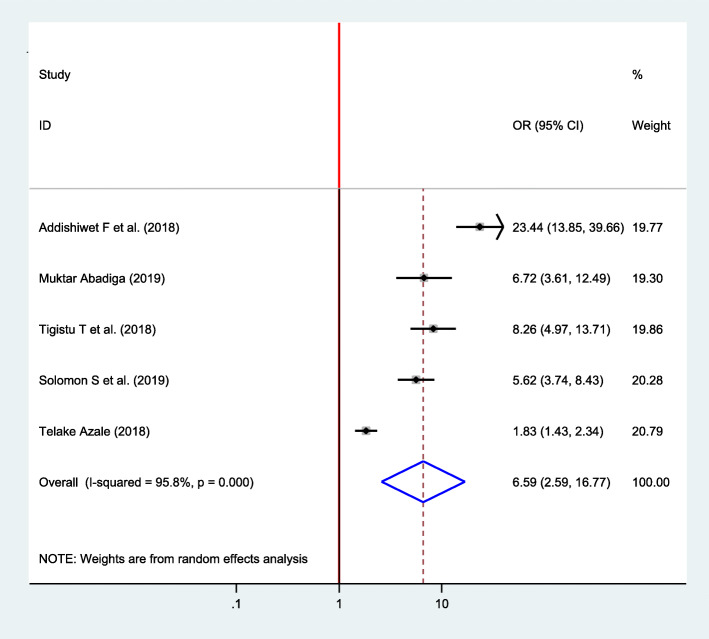
Forest plot for pooled association between social support and postpartum depression in Ethiopia, 2020

Five studies were included to observe the association between social support and postpartum depression [15, 22, 23, 33, 37]. Accordingly, five of the included studies indicated a significant association between social support and postpartum depression. The pooled result showed that postpartum depression was 6.59 times higher among women who lack social support as compared with women who had social support (OR = 6.59, 95% CI 2.59, 16.77) (Fig. 10).

#### Association between the history of infant death and postpartum depression

**Fig. 11:**
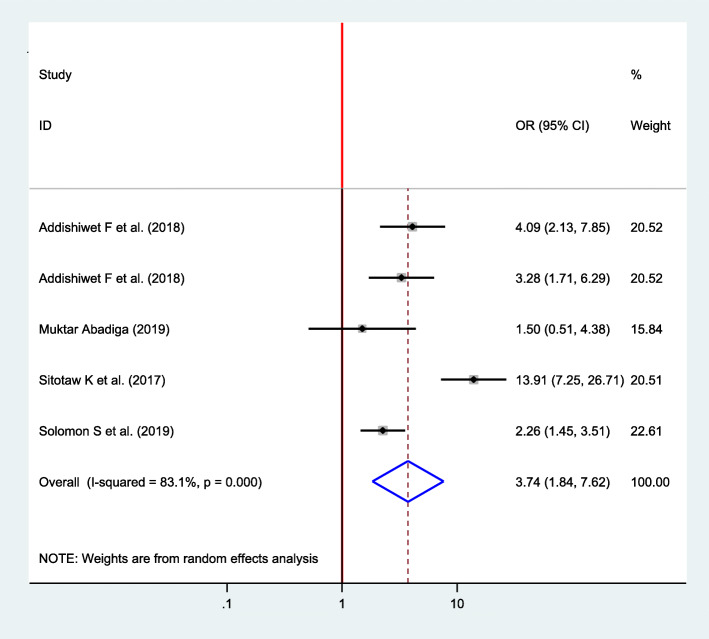
Forest plot for pooled association between history of infant death and postpartum depression in Ethiopia, 2020

Five articles were included in analysis to determine the association between the history of infant death and postpartum depression [16, 22, 33, 34, 37], of which four of them showed a significant association [16, 33, 34, 37]. The pooled finding showed that the odds of developing postpartum depression among women who had experienced previous history of infant death was 3.74 times higher as compared with their counterparts (OR = 3.74, 95% CI 1.84, 7.62) (Fig. 11).

#### Association between marital satisfaction and postpartum depression

**Fig. 12:**
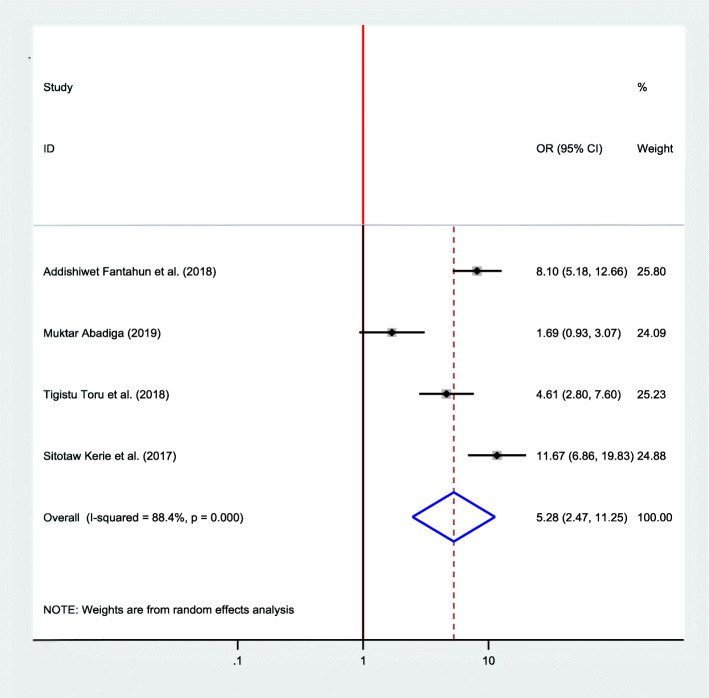
Forest plot for pooled association between marital satisfaction and postpartum depression in Ethiopia, 2020

Four studies were included in the final meta-analysis to determine the pooled effect of marital satisfaction on postpartum depression [16, 22, 23, 33]. Since high heterogeneity was observed (*I*^2^ = 88.4, *P* value < 0.001), a random effects model was used. The finding discovered that the odds of developing postpartum depression was 5.28 times more likely among women who had no marital satisfaction than their counterparts (OR = 5.28, 95%, CI 2.47, 11.25) (Fig. 12).

## Discussion

The meta-analysis of twelve cross-sectional studies, including 9674 mothers derived 22.89% a national pooled estimate of postpartum depression. A systematic review and meta-analysis in India and Turkey found a comparable pooled prevalence of postpartum depression which is 22% and 23.8%, respectively [39, 40]. However, this finding is higher than the global magnitude of PPD, which is 17% [41]. This may be due to the previous review which included fewer studies from Africa. And, the current review estimate of PPD was also higher than a report from a meta-analysis of 291 studies from 56 countries [42]. The discrepancy might be due to the setting where the studies were conducted. In the previous study, most of the studies included in the review were from a developed country, and the magnitude of PPD may be lower in a developed country than a low-income country due to economic and health disparities. In addition, the management and screening method of PPD during postpartum period is better in a developed country than a developing country.

However, this finding is slightly lower than a study conducted in Bangladesh [11] and previously conducted evidence in Ethiopia [43]. This might be due to the study conducted in Bangladesh that uses a purposive sampling technique and a small sample size which may overestimate the magnitude of PPD. The discrepancy from another evidence might be due to the review focused on perinatal depression, which reports the cumulative depression burden in women during pregnancy, and postpartum period while the current evidence is specifically focused on postpartum depression.

The current meta-analysis also revealed disparities across regions of the country. The highest and lowest prevalence were observed in the SNNP region. This could be due to the variation across population characteristics, living conditions, and sample size. It is evident that there are discrepancies on the estimate of the prevalence of PPD across the nation and within-country [42].

It was evidenced that there is no single cause or risk factor for PPD; reasonably, the etiology of PPDs is multifactorial. The risk of PPD was found to be fourfold higher among women who had the previous history of depression than their counterparts. This finding is in agreement with a wide-reach review, which reveals the previous history of depression as a strong risk factor for PPD [1]. Another study also reported a strong positive association between postpartum depression and previous history of depression [43, 44]. The similarity could be explained by most risk factors for depression during pregnancy, childbirth and postpartum period are common. Therefore, women who had the previous history of depression need special attention to reduce the risk of PPD.

It is known that the risk of developing depression throughout the process of pregnancy, childbirth, and postnatal period in women who are not living with their spouse is higher [43]. The current pooled estimate also evidenced that mothers who had social support were less likely to develop postpartum depression than those who lack social support. It is also known that lack of psychological and economic support from social were recurrent risk factors for the development of depression [1, 39]. Lack of social support either perceived or received was also found to be a strong risk factor for PPD [1]. In addition, the current finding is scientifically supported by a study conducted in Ireland in which formal structural, functional, and emotional support were found to be significant predictors of PPD [45]. This could be explained by a finding from another meta-analysis, which verified peer support is better than the usual care in reducing depressive symptoms during the postnatal period [46]. Therefore, antenatal screening for high-risk women would help to prevent PPD, and home visits after childbirth would minimize the burden of PPD because the women perceives she has someone to talk about anything.

Women who had unplanned pregnancy were more likely to develop postpartum depression. This finding is in accordance with another meta-analysis report [39]. A study conducted in Bangladesh and Malaysia also identified unplanned pregnancy as a significant risk factor for PPD [11, 47]. Other evidence also found the risk of depression is threefold higher among mothers who had unplanned pregnancy [43]. This may be a result from inadequate preparation for childbirth and poor coping mechanism to combat inherent stressors during pregnancy, childbirth, and the postpartum period.

The current review found no statistically significant association between the type of delivery and PPD. However, this finding is inconsistent with another meta-analysis, which reports increased risk of postpartum depression among women who gave birth by cesarean section [48]. This may be due to a small sample size in the current review as the previous study included a large number of studies.

Abusive marital relationship is a well-known stressor during the postnatal period [49]. The result of the current review also showed a positive association between marital dissatisfaction and postpartum depression. This finding is in agreement with another study that reports a significant inverse association between marital satisfaction and PPD [50]. This may be due to women with a new baby who may seek additional love and support from her partner. In addition to the marital relationship, living with a spouse cannot be a guarantee for social support because partner violence was also found to increase PPD [11, 43, 47, 49]. Another finding also reported a positive association between PPD and domestic violence [51]. The current estimate is also in accordance with this finding. Women who experience domestic violence were more likely to develop postpartum depression. This similarity might be due to domestic violence, especially in the Ethiopian setting where it is a silent private situation in most cultures. The community also accepts partner violence as a traditional norm, and even the legal bodies or police stations engage as a mediator rather than enforcing laws.

Women who had no history of infant death were less likely to develop postpartum depression. This finding is supported by another study which revealed the experience of the death of infant increases the risk of postpartum depression [11].

### Strengths and limitations of the study

This review has several strengths including the following: this review tried to identify all potential determinants of postpartum depression. Moreover, we used comprehensive search strategies and PRISMA checklist to improve the quality of the review. All published and unpublished studies are involved in the analysis. Whereas, this review has limitations such as the review included studies that were published only in the English language. Also, the other limitation of this review is that the protocol of this manuscript was not registered on PROSPERO. Finally, all of the included studies in the final analysis were cross-sectional study designs which may decrease causal conclusion between the outcome variable and its determinants.

## Conclusions and recommendations

We found a higher prevalence of postpartum depression. Domestic violence, absence of social support, marital dissatisfaction, history of infant death, unplanned pregnancy, and previous history of depression were independent determinants of postpartum depression in Ethiopians. Limited access to effective PNC may contribute to most determinants of PPD. Therefore, we would like to recommend integrated mental health care during postnatal care service. The time after giving birth is stressful especially for new mothers, so emotional and psychosocial support should be provided at the community level as well as at health care settings to reduce the risk of depression. Postnatal depression can result in adverse public health problems. Most determinants identified by this review can be managed by a continuum of maternal and child health care. Therefore, strengthening the postnatal care program should be considered to minimize the burden of PPD.

## Data Availability

All data analyzed during this study are included in the manuscript.

## Abbreviations

PNC: Postnatal care
PPD: Postpartum depression
CI: Confidence interval
EPDS: Edinburgh Postnatal Depression Scale
OR: Odds ratio
SNNP: Southern Nation Nationalities and People

## References

[1] Stewart DE, Robertson E, Dennis CL, Grace SL, Wallington T (2003). Postpartum depression: literature review of risk factors and interventions.

[2] Rai S, Pathak A, Sharma I (2015). Postpartum psychiatric disorders: early diagnosis and management. Indian J Psychiatry.

[3] Amercian Psychiatric Association. Diagnostic and Statistical Manual of Mental Disorders, Fourth Edition. Washington, DC: American Psychiatric Press; 1994.

[4] Janca A, Hiller W (1996). ICD-10 checklists—a tool for clinicians' use of the ICD-10 classification of mental and behavioral disorders. Compr Psychiatry.

[5] Affonso DD, De AK, Horowitz JA, Mayberry LJ (2000). An international study exploring levels of postpartum depressive symptomatology. J Psychosom Res.

[6] Gelaye B, Rondon MB, Araya R, Williams MA (2016). Epidemiology of maternal depression, risk factors, and child outcomes in low-income and middle-income countries. Lancet Psychiatry.

[7] Gavin NI, Gaynes BN, Lohr KN, Meltzer-Brody S, Gartlehner G, Swinson T (2005). Perinatal depression: a systematic review of prevalence and incidence. Obstet Gynecol.

[8] Liu S, Yan Y, Gao X, Xiang S, Sha T, Zeng G, He Q (2017). Risk factors for postpartum depression among Chinese women: path model analysis. BMC pregnancy and childbirth.

[9] Ikeda M, Kamibeppu K (2013). Measuring the risk factors for postpartum depression: development of the Japanese version of the Postpartum Depression Predictors Inventory-Revised (PDPI-RJ). BMC pregnancy and childbirth.

[10] Hegde S, Latha KS, Bhat SM, Sharma PS, Kamath A, Shetty A (2012). Postpartum depression: prevalence and associated factors among women in India. J Womens Health Issues Care.

[11] Azad R, Fahmi R, Shrestha S, Joshi H, Hasan M, Khan AN, Chowdhury MA, El Arifeen S, Billah SM (2019). Prevalence and risk factors of postpartum depression within one year after birth in urban slums of Dhaka, Bangladesh. PLoS One.

[12] Ongeri L, Wanga V, Otieno P, Mbui J, Juma E, Vander Stoep A, Mathai M (2018). Demographic, psychosocial and clinical factors associated with postpartum depression in Kenyan women. BMC Psychiatry.

[13] Anokye R, Acheampong E, Budu-Ainooson A, Obeng EI, Akwasi AG (2018). Prevalence of postpartum depression and interventions utilized for its management. Ann General Psychiatry.

[14] Khalifa DS, Glavin K, Bjertness E, Lien L (2016). Determinants of postnatal depression in Sudanese women at 3 months postpartum: a cross-sectional study. BMJ Open.

[15] Azale T, Fekadu A, Hanlon C (2018). Postpartum depressive symptoms in the context of high social adversity and reproductive health threats: a population-based study. Int J Ment Heal Syst.

[16] Kerie S, Menberu M, Niguse W (2018). Prevalence and associated factors of postpartum depression in Southwest, Ethiopia, 2017: a cross-sectional study. BMC research notes.

[17] Lester SW, Turnley WH, Bloodgood JM, Bolino MC (2002). Not seeing eye to eye: Differences in supervisor and subordinate perceptions of and attributions for psychological contract breach. J Organ Behav.

[18] Corey E, Thapa S (2011). Postpartum depression: an overview of treatment and prevention.

[19] Rahman A, Harrington R, Bunn J (2002). Can maternal depression increase infant risk of illness and growth impairment in developing countries?. Child Care Health Dev.

[20] Anoop S, Saravanan B, Joseph A, Cherian A, Jacob KS (2004). Maternal depression and low maternal intelligence as risk factors for malnutrition in children: a community based case-control study from South India. Arch Dis Child.

[21] Dennis CL (2005). Psychosocial and psychological interventions for prevention of postnatal depression: systematic review. Bmj..

[22] Abadiga M. Magnitude and associated factors of postpartum depression among women in Nekemte town, East Wollega zone, west Ethiopia, 2019: A community-based study. PLoS One. 2019;14(11). 10.1371/journal.pone.0224792 PMC6853315 31721808

[23] Toru T, Chemir F, Anand S (2018). Magnitude of postpartum depression and associated factors among women in Mizan Aman town, Bench Maji zone, Southwest Ethiopia. BMC pregnancy and childbirth.

[24] Shidhaye PR, Giri PA (2014). Maternal depression: a hidden burden in developing countries. Annals of medical and health sciences research.

[25] Teshome H. Postpartum depression and associated factors among mothers: the case of Nifas Silk Lafto Sub City Woreda 1 and 2 health centers (Doctoral dissertation, Addis Ababa University), Addis Ababa; 2016.

[26] Fantahun A (2016). Prevalence and associated factors of postpartum depression among mothers attending puublic health centers of Addis Ababa.

[27] Christi L. Gross. Maternal age and postpartum depression during the transition to parenthood (Doctoral dissertation, Kent State University), Kent State University; 2016.

[28] Shelton SL. Postpartum depressive symptoms: a study of influencing factors and an intervention for improvement; 2015.

[29] Santos IS, Matijasevich A, Tavares BF, Barros AJ, Botelho IP, Lapolli C, Magalhães PV, Barbosa AP, Barros FC (2007). Validation of the Edinburgh Postnatal Depression Scale (EPDS) in a sample of mothers from the 2004 Pelotas Birth Cohort Study. Cadernos de Saúde Pública.

[30] Löwe B, Unützer J, Callahan CM, Perkins AJ, Kroenke K (2004). Monitoring depression treatment outcomes with the patient health questionnaire-9. Med Care.

[31] Andrews G, Slade T (2001). Interpreting scores on the Kessler psychological distress scale (K10). Aust N Z J Public Health.

[32] Shewangzawa A, Tadesse B, Ashani T, Misgana T, Shewasinad S. Prevalence of postpartum depression and associated factors among postnatal women attending at Hiwot Fana Specialized University Hospital, Harar, East Ethiopia.

[33] Adamu AF, Adinew YM (2018). Domestic violence as a risk factor for postpartum depression among Ethiopian women: facility based study. Clinical practice and epidemiology in mental health: CP & EMH.

[34] Fantahun A, Cherie A, Deribe L (2018). Prevalence and factors associated with postpartum depression among mothers attending public health centers of Addis Ababa, Ethiopia, 2016. Clinical practice and epidemiology in mental health: CP & EMH.

[35] Abebe A, Tesfaw G, Mulat H, Hibdye G (2019). Postpartum depression and associated factors among mothers in Bahir Dar Town, Northwest Ethiopia. Ann General Psychiatry.

[36] Upadhyay AK, Singh A, Singh A. Association between unintended births and risk of postpartum depression: Evidence from Ethiopia, India, Peru and Vietnam. SSM-population health. 2019;9:100495. 10.1016/j.ssmph.2019.100495 PMC6804781 31650000

[37] Shitu S, Geda B, Dheresa M (2019). Postpartum depression and associated factors among mothers who gave birth in the last twelve months in Ankesha district, Awi zone, North West Ethiopia. BMC Pregnancy Childbirth.

[38] Deribachew H, Berhe D, Zaid T, Desta S (2016). Assessment of prevalence and associated factors of postpartum depression among postpartum mothers in eastern zone of Tigray. Eur J Pharm Med Res.

[39] Özcan NK, Boyacıoğlu NE, Dinç H (2017). Postpartum depression prevalence and risk factors in Turkey: a systematic review and meta-analysis. Arch Psychiatr Nurs.

[40] Upadhyay RP, Chowdhury R, Salehi A, Sarkar K, Singh SK, Sinha B, Pawar A, Rajalakshmi AK, Kumar A (2017). Postpartum depression in India: a systematic review and meta-analysis. Bull World Health Organ.

[41] Shorey S, Chee CY, Ng ED, Chan YH, San Tam WW, Chong YS (2018). Prevalence and incidence of postpartum depression among healthy mothers: a systematic review and meta-analysis. J Psychiatr Res.

[42] Hahn-Holbrook J, Cornwell-Hinrichs T, Anaya I (2018). Economic and health predictors of national postpartum depression prevalence: a systematic review, meta-analysis, and meta-regression of 291 studies from 56 countries. Front Psychiatry.

[43] Mersha AG, Abebe SA, Sori LM, Abegaz TM (2018). Prevalence and associated factors of perinatal depression in Ethiopia: a systematic review and meta-analysis. Depress Res Treat.

[44] Nasreen HE, Edhborg M, Petzold M, Forsell Y, Kabir ZN (2015). Incidence and risk factor of postpartum depressive symptoms in women: a population based prospective cohort study in a rural district in Bangladesh. J Depress Anxiety.

[45] Leahy-Warren P, McCarthy G, Corcoran P (2011). Postnatal depression in first-time mothers: prevalence and relationships between functional and structural social support at 6 and 12 weeks postpartum. Arch Psychiatr Nurs.

[46] Pfeiffer PN, Heisler M, Piette JD, Rogers MA, Valenstein M (2011). Efficacy of peer support interventions for depression: a meta-analysis. Gen Hosp Psychiatry.

[47] Ahmad NA, Silim UA, Rosman A, Mohamed M, Chan YY, Kasim NM, Yusof M, Abd Razak MA, Omar M, Aziz FA, Jamaluddin R. Postnatal depression and intimate partner violence: a nationwide clinic-based cross-sectional study in Malaysia. BMJ open. 2018;8(5). 10.1136/bmjopen-2017-020649 PMC5961592 29764882

[48] Xu H, Ding Y, Ma Y, Xin X, Zhang D (2017). Cesarean section and risk of postpartum depression: a meta-analysis. J Psychosom Res.

[49] Hanlon C, Whitley R, Wondimagegn D, Alem A, Prince M (2009). Postnatal mental distress in relation to the sociocultural practices of childbirth: an exploratory qualitative study from Ethiopia. Soc Sci Med.

[50] Odinka JI, Nwoke M, Chukwuorji JC, Egbuagu K, Mefoh P, Odinka PC, Amadi KU, Muomah RC (2018). Post-partum depression, anxiety and marital satisfaction: a perspective from Southeastern Nigeria. S Afr J Psychiatry.

[51] Wu Q, Chen HL, Xu XJ (2012). Violence as a risk factor for postpartum depression in mothers: a meta-analysis. Archives of women's mental health.

